# Novel neural network application for bacterial colony classification

**DOI:** 10.1186/s12976-018-0093-x

**Published:** 2018-12-02

**Authors:** Lei Huang, Tong Wu

**Affiliations:** 1 Department of Clinical Laboratory, Peking University First Hospital, 8 Xishiku Street, Beijing, China; 2 Peking University Health Science Center, 38 Xueyuan Road, Beijing, China

**Keywords:** Bacterial colony, Classification, Convolutional neural network, Clinical laboratory

## Abstract

**Background:**

Bacterial colony morphology is the first step of classifying the bacterial species before sending them to subsequent identification process with devices, such as VITEK 2 automated system and mass spectrometry microbial identification system. It is essential as a pre-screening process because it can greatly reduce the scope of possible bacterial species and will make the subsequent identification more specific and increase work efficiency in clinical bacteriology. But this work needs adequate clinical laboratory expertise of bacterial colony morphology, which is especially difficult for beginners to handle properly. This study presents automatic programs for bacterial colony classification task, by applying the deep convolutional neural networks (CNN), which has a widespread use of digital imaging data analysis in hospitals. The most common 18 bacterial colony classes from Peking University First Hospital were used to train this framework, and other images out of these training dataset were utilized to test the performance of this classifier.

**Results:**

The feasibility of this framework was verified by the comparison between predicted result and standard bacterial category. The classification accuracy of all 18 bacteria can reach 73%, and the accuracy and specificity of each kind of bacteria can reach as high as 90%.

**Conclusions:**

The supervised neural networks we use can have more promising classification characteristics for bacterial colony pre-screening process, and the unsupervised network should have more advantages in revealing novel characteristics from pictures, which can provide some practical indications to our clinical staffs.

## Background

The rapid development of computational imaging processing systems has largely benefited the hospitals for image analysis in diagnosis and investigation. Most of these technologies are applied to facilities such as usage in iconography: ultrasonography, CT, SPECT and MRI [1–4], but few of them has been used directly as a computational vision method in bacterial classification. While there are some limitations of the traditional bacterial classification methods which depend on clinical expertise in very diverse colony structures of different bacterial species [5]. Firstly, they are time-consuming and laborious for staffs [6]. Secondly, the identification needs enough morphology expertise of bacterial colonies, which is difficult for beginners to tackle with. Therefore, the development of an automatic bacterial colony morphology identification system is helpful for aiding clinical staffs in reducing workload as well as acting as a reference for beginners. Some literatures have presented the useful features to identify different bacterial species such as shape, size, surface, border, opacity and color [7], with the combination of staining methods, such as Gram Staining [8]. While the discrimination of bacterial strains from a large amount of samples is still very complicated. The traditional manual discriminations based on expert classifying experience of circular or irregular colony shape, convex or concave colony elevation, etc., require lots of manpower and clinical expertise, especially in the situation with large amount of samples in a tertiary hospital. And a framework based on bacterial colony morphology data can be a convenient way for supplementing the preliminary identification process. It is really necessary to build up this computational vision framework, which can automatically classify lots of bacteria as the preliminary screen preparation results. These results are able to be utilized to narrow down the classification scope in the following more advanced and specific identification procedure such as utilization of VITEK 2 system [9] and MALDI-TOF mass spectrometry detection system [10].

Deep learning system is a kind of machine learning method based on learning data representations [11], and one of its applications is computational vision. For computational vision, it mimics the arrangement of neural network in human brain, which can learn and transport different qualities of information through multiple layers of transformation [12]. It can get feature representations by automatically learning from raw images without applying human experience, and it can model these abstract features by using constituent multiple non-linear transformations. The convolutional neural network is one of these machine learning methods that mimics the connectivity pattern between neurons of visual cortex [13]. It can extract hierarchical image feature representations based on multi-layer processing. And the features extracted are able to have a better performance than that from hand-crafted features because they are more general and less subjective.

In this study, feature representations of colonies from 18 bacterial species were learnt to build convolutional neural network. Commonly, preliminary bacterial classification was given out as training labels by human experience of bacterial colonies in clinical laboratory. Then these labels could be aligned with specific features of bacterial species extracted from our networks, which could be recorded and be further applied for our networks prediction. The building of one computational classification model depended on supervised images with standard labels, while the building of the other model relied on unsupervised images which were only for this neural network to extract colonial features. The predicted labels and annotations from both neural networks are quite useful because they can provide some aids for the operation of clinical laboratory and they are able to show inspirations of bacterial colony classification to clinical staffs based on their extracted features.

## Methods

The following image classification techniques are divided into supervised and unsupervised classifications. The supervised classification methods include a traditional convolutional neural network and a special convolutional neural network named AlexNet designed by the SuperVision group for large scare visual recognition [14]. Besides the input layer and output layer, both these two CNN models are generally consisted of four types of layers, which is convolutional layer, ReLU layer, pooling layer and fully connected layer. The function of each layer and detailed processes of training are given in the following part. Then, a kind of unsupervised method named Autoencoder is introduced, and its general structure and detailed training processes are presented, to make comparison with the supervised methods and to verify the feasibilities of both methods.

### Traditional convolutional neural network

**General structure:** Our convolutional neural network has basic architecture built with multiple distinct layers, having their own unique functions to transform input volumes to output volumes. Besides the input and output layer, there are some hidden layers in between that play the most important part in filtering and transporting information. These are convolutional layer, pooling layer and fully connected layer. Convolutional layer has a set of learning filters, and each filter has many receptive fields that expand through the whole filter of the input. Depth of a convolutional layer is the number of neurons in a layer, and each neuron is able to extract a specific feature detected from positions of the input. A 2-dimensional feature map is constructed based on these features, and it will have spatially local correlation with neurons of next adjacent layer by receptive fields, a spatially connectivity pattern. Thus the feature map will act as the input of the next layer, and each output volume can be represented as a neuron output that projects to a small region in the input. In this way, spatial image information is extracted and transported by multiple connective layers. While because of so many parameters and large size of the convolutional network representation, pooling layer is proposed to reduce both values to decrease computational load as well as avoid over-fitting. During the processing of pooling, the number of neurons in each layer, the depth of dimension remains unchanged, only the size of each depth slide will be reduced. Finally, at the end of some convolutional layers and interspersed several pooling layers, all activations are gathered in one layer, the fully connected layer. Considering the input signal, weight and bias together, this fully connected layer can generate the output value. Depending on this, fully connected layer can do the high-level judgement for bacterial classification.

**Fig. 1:**
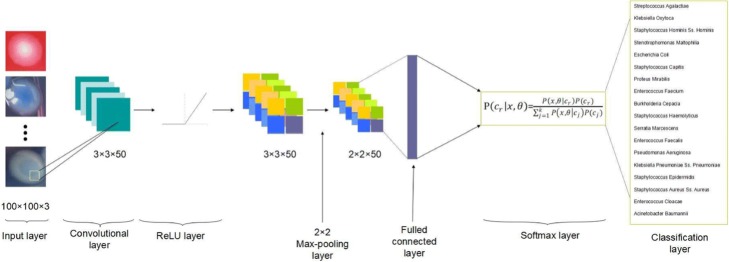
The general structure of the conventional neural network

**Each layer:** In our convolutional network classification model, there are totally seven layers which represent different functions successively, and the general scheme of these connected seven layers network is depicted in Fig. 1. There are image input layer, convolutional layer, rectified linear layer, max pooling layer, fully connected layer, softmax layer and classification layer, respectively.

The first layer is the image input layer, which determines the size, the height and width, of each input image. The point is that the size of each image must be identical in the 3 dimensional scopes. The three dimensions represent the height, width, and RGB color of images, respectively. And here as our input images are colored ones which need the color channel to be set at 3, our input set is 100-by-100-by-3. The next layer is the convolutional layer, and its parameters consist of several filters, which are convolved across the height and width of the input parameter of each image. The size of filter is very important because it determines the height and width the filter utilizes to screen the whole image. Here we use the size 3 which means the size of filters we applies in the convolutional layer is 3-by-3. We choose this small scope of size because the discriminations among different kinds of bacteria is subtle, such as different shape of margin of colonies, for the reason that small scopes of filters can have a better characteristics to highlight the fine variations among bacterial colonies. To fully represent all features of the bacterial colony image, we set the number of filter as 50, which means that there are equivalently 50 neurons that can extract, evaluate and transport 50 kinds of information into the next layer of visualization. When the input volume of images has been transported through the convolutional layer, the dot products are calculated between the entries of filters and the input. From the dot product map, the convolutional layer can detect some specific features at some spatial spaces in the input layer, and these detected features will serve as an activation of filters. We combine all the activated filters together to activation maps, which are able to act as the output volume of this convolutional layer.

The following layer of the convolutional layer is the ReLU layer, which is for rectifying the convolutional output volume by linear function. The processing function in this layer can be written as *f*(*x*)=*x*^+^=*max*(0,*x*), which can help remove negative values in the convolutional output and do linear amplification to positive values [15].

We set a pooling layer after processing of the rectified linear unit. It is responsible for reducing the spatial dimension from the output of previous convolutional layer, and it aims at decreasing computational overhead and load as well as avoiding over-fitting for lots of parameters to consider. Here the pooling method we utilize is the max pooling algorithm, which can screen the input image with rectangular region and then figure out the maximum element in this region. The height and width of this screening rectangular region is 2, and we also set the stride, number of elements for moving along the image horizontally and vertically, as 2. In this way, as the dimension of stride being equal to that of the screening region, in every period of screening there will be no overlap between these screening regions.

The following fully connected layer will wholly connects to all filters, all neurons, in the previous layer. It can combine all the features learned from previous layers and integrate these information for overall consideration of classification. Here our output size of this layer is 18, that means there are 18 kinds of classification being figured out by the fully connected layer’s information integration. When there are more than two classifications in consideration, the output value of the fully connected layer would be activated by a softmax layer’s function. Assume there are totally k classifications in this project, and we denote the set of observed data by *x* and the set of parameters by *θ*. The recognized probability of each classification would be:

1 $$ P(c_{r}|x,\theta)=\frac{P(x,\theta|c_{r})P(c_{r})}{{\sum\nolimits}_{j=1}^{k}P(x,\theta|c_{j})P(c_{j})}  $$

Here *P*(*c*_*r*_) is the prior probability of class r, and the ${\sum \nolimits }_{j=1}^{k}P(c_{j}|x,\theta)$ is equal to 1 [16]. Then the returned probabilities by this activation function will be assigned to each exclusive class by final classification layer.

**Detailed training:** The set of parameters for training and the selection of training algorithm are essential for the feasibility of this model building and application. Here we set the algorithm of training as stochastic gradient descent with momentum, the number of circles on the whole training data as 30, and the initial learning rate as 0.0001. When we get the error function that is calculated from the entire training data and the output training prediction after several training iterations, the stochastic gradient descent algorithm is able to minimize the error functions by taking steps in the fastest gradient descent directions [17]. We assume one parameter of the training model is *a*, the calculated error function is *E*(*a*), and the learning rate is *μ*, thus the i+1 times of modification of this parameter can be expressed as:

2 $$ a_{i+1}=a_{i}-\mu\nabla E(a_{i})  $$

And a momentum term, which is how much the parameter from previous step contributes to current iteration, can be added to this equation so as to prevent the error function E(a) from oscillating around several steepest descents to the objective function [18]. Assume the *σ* as the coefficient of contribution from parameter in previous iterations to the current iterations, so the modified function can be written as:

3 $$ a_{i+1}=a_{i}-\mu\nabla E(a_{i})+\sigma(a_{i}-a_{i-1})  $$

This momentum term can be explained from an analogy to momentum in physics, that would cause acceleration from the gradient of the force, so as to travel in the direction which is able to reduce oscillation [19].

Another issue is that we have 18 classifications and 18 dataset of observations, and the whole error function *E*(*a*) can be divided into the sum of error functions from each dataset, $\frac {1}{k}\sum _{j=1}^{k}E(a_{j})$. In this way the parameters can be updated by minimizing the whole error function through stochastic gradient decent algorithm, and calculating the gradients of the parameter of current iteration with backpropagation algorithm. By getting these gradients as input and updating them to minimize the error functions again and again, the neural network training model can be built to mimic the entire training data in an optimal way.

### AlexNet neural network

**General structure:** For the reason that our first convolutional neural network is simple, to achieve a more complicated task of classifying 18 bacteria colonies with similar morphologies, a bigger and deeper convolutional neural network needs to be considered. Here we adopt a pretrained famous convolutional neural network, the AlexNet neural network, to solve this classification problem. As AlexNet neural network is a kind of convolutional neural network, the general structure of this network also consists of image input layer, convolutional layer, pooling layer, fully connected layer, and etc. However, the AlexNet neural network is much deeper compared with common convolutional neural network, which has more channels of convolutional layers and gathering layers of these multi-channel layers to collect and normalize information [20]. And these specific layers of AlexNet neural network can be called cross channel normalization layer. What is more, as the distributions of convolutional layers are different, there are many convolutional layers from different channels set at the second layer of the AlexNet so as to extract more features from images, and after a set of pooling layers there will be a series of dense convolutional layers, that is these featured convolutional layers will stack on top of each other without any pooling layer inserting in the middle [21].

**Each layer and detailed training:** We have a total of 25 layers in this AlexNet architecture. The first layer is the image input layer for dimensions of 227×227×3 images. The next are two convolutional-ReLU-cross channel normalization-pooling layers’ circles, with following condensed convolutional layers and ReLU layers. The dimensions of convolutional layers in the first two circles are 11×11×3,5×5×48, and 3×3×256, respectively. And dimensions of subsequent dense convolutional layers are 3×3×192,3×3×192 in order. The following are fully connected layers with insertions of ReLU layers and dropout layers. In the end, they are softmax layer and classification layer, which are similar with that in our previous convolutional neural network. The dropout layer in this AlexNet architecture is to dropout half of the trained neurons randomly, which is responsible for reducing the computational load, especially for this multi-channel convolutional neural network, but it will not decrease any parameters calculated from the training process. The multi-channel design coupled with dropout method will effectively prevent over-fitting and long computation time. That is also an essential improvement of the AlexNet to deal with large amount of training data.

The application of this AlexNet network is not to train the network from the very beginning, but to adapt it to our application. Thus after getting the training data from half of the entire data, we choose the 20th layer, the second fully connected layers array, of the network to extract features from our training image data. Because the layer array we choose is the 4096-element fully connected layer, we can get a 4096-element feature from each image, and we set this 4096×2491 feature matrix as the *trainingfeatures* matrix. Next, for this supervised learning model to be built, we label every image from the 2491 with their standard bacterial categories. By combing the *trainingfeatures* matrix and the labeled categorical matrix together, we can build a support vector machine (SVM) classifier. This algorithm can efficiently perform a non-probabilistic binary linear classification as well as a non-linear classification with kernel trick. Based on qualities of AlexNet network and SVM algorithm, a complicated and efficient classifier which is suitable to deal with large amount of data and more classifications is built.

### Unsupervised Autoencoder neural network

**General structure:** The morphology variance of bacterial colonies is great, especially for images of bacterial colonies cultured in different agar plates, such as maconkey agar and blood agar. To construct models that can find patterns more accurate and can tolerate anomalies, we use both supervised and unsupervised machine learning methods to tackle with this classification problem. For the unsupervised artificial neural network, we apply the Autoencoder neural network, which can extract features automatically with unlabeled bacterial colony images.

Our Autoencoder has one input layer, one output layer, and some hidden layers connected between them. The number of nodes in the input layer is the same as that in the output layer, with a feedforward, non-recurrent training direction. Another feature of this Autoencoder neural network different from convolutional neural network are the encoder and decoder parts [22]. In this case, every input value *x* from input space will be weighted with a weight matrix *W* to get the code:

4 $$ z=\alpha(Wx+\delta)  $$

Here *z* is the corresponding code, the latent variable is *x*, *α* is the activation function for this input, and *δ* is the bias vector. After encoding, the decoder layer would transfer the *z* map to the structure of output space of $\acute x$, which has the same dimension as the input space of *x*.

5 $$ \acute x=\acute\alpha\left(\acute Wz+\acute\delta\right)  $$

And by backpropagation algorithm, each $\acute x$ value from output space will be made similar to the *x* value from input space by adjusting the identity function. Thus the error function of *x* and $\acute x$ can be built as [23]:

6 $$ E(x,\acute x)=\left|x-\acute\alpha(\acute W(\alpha(Wx+\delta))+\acute\delta)\right|^{2}  $$

By minimizing the error function and updating the weights, this model can improve its performance continuously.

**Each layer and detailed training:** There are several components for the model constitution. The hidden units in the first hidden layer are 800, which is forced to learn a compressed features from the input. The dimension of our input image 50×50×3 is much larger than 800, which makes the encoder layer be able to learn more significant features for the bacterial colonies discrimination. The training epochs we set in the first hidden layer, in the encoder layer is 400, the weight of each element in the input space as we describe above is 0.004, and the average output of each neuron in the hidden layer is 0.15. After encoding, the decoder layer will try to reverse the mapping to reconstruct the original input.

After getting the extracted data from the hidden layer of the first Autoencoder model, we can build a second Autoencoder model with the similar way. The difference of this second Autoencoder is that it does not utilize the training data but taking use of the features from the first Autoencoder model instead. The input size of the second Autoencoder model is even smaller, and it can give out smaller representative features of the colonies. Here the dimension we reduce by the second Autoencoder is 200, and the final layer is the 18-dimensional layer aiming to classify these 200-dimensional vectors into 18 digit classes.

The final layer is the softmax layer. It will use the 200 extraction features from the second Autoencoder to train with the label matrix, and the epochs of training is 400. Finally, we use these above models to form a stack neural network in order. We apply our test images into this stack neural network and visualize the accuracy with a confusion matrix. We can also use backpropagation manually to improve the tuning of this multilayer network, and it can be seen from the statistical results that the performance becomes much better.

## Results and discussion

In this paper, three popular neural network algorithms have been used for training and testing the bacterial colony classification models. The feasibility of these proposed models has been evaluated in terms of the standard classification of bacterial images from clinical database.

### Dataset

**Fig. 2:**
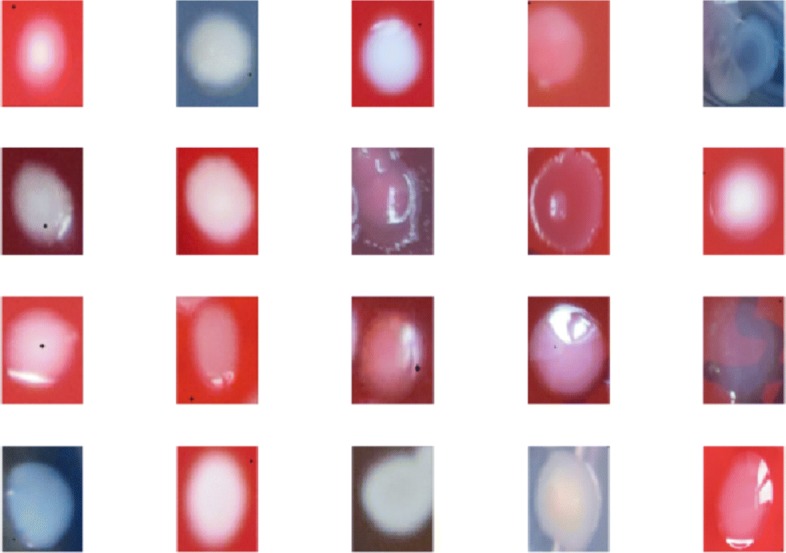
Images from each bacterial class showing the interclass variations

**Fig. 3:**
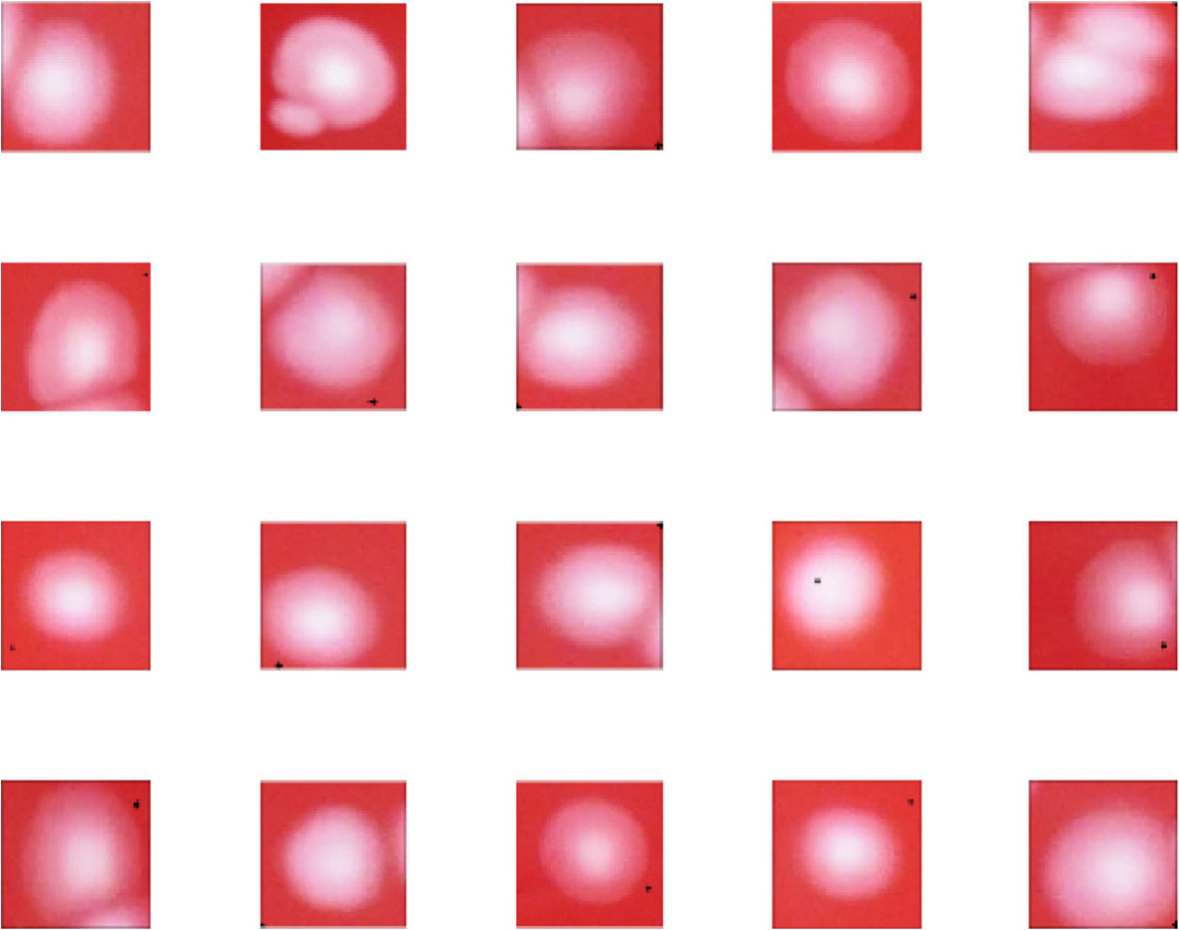
Images from *Streptococcus agalactiae* class showing the intra variations

The utilized dataset for these proposed models has been collected from clinical microbiology lab of Peking University First Hospital and classes are given based on the species of these bacterial, such as *Escherichia coli*, *Klebsiella pneumonia*, *Staphylococcus aureus*, *Pseudomonas aeruginosa* and etc. There are totally 18 classes of bacterial colonies in this dataset, which all belong to the most common human pathogenic bacteria. The total number of every bacterial class in this dataset is 4982. And the images from each class have been divided into both training and testing sets. The percentages of images for training set and testing set are both 50%. 2491 images are used in training set and 2491 are used in testing set, respectively. The input images for the convolutional neural network and the AlexNet neural network have the 100×100 dimensions with colors, and the input images for the unsupervised Autoencoder neural network have the dimension of 50×50×3, which would greatly reduce the computational load for this model. And some examples are given in this paper shown as Figs. 2 and 3 which represents the differences among different classifications and variations among individual images of each class.

### Classification performance

To evaluate the performance of these three models, we use several parameters, the true positive *T*^+^, the false positive *F*^+^, the true negative *T*^−^ and the false negative *F*^−^. The sensitivity, is the model to correctly classify the bacterial colonies into their species, can be expressed as [24]:

7 $$ Sensitivity=\frac{1}{18}\sum_{j=1}^{18}\frac{T^{+}}{T^{+}+F^{-}}  $$

The specificity for correctly rejecting bacteria to classifications that are not belonging to, has the expression as:

8 $$ Specificity=\frac{1}{18}\sum_{j=1}^{18}\frac{T^{-}}{T^{-}+F^{+}}  $$

And the precision and accuracy of each model have the expression: $Precision=\frac {1}{18}\sum _{j=1}^{18}\frac {T^{+}}{T^{+}+F^{+}}$ and $Accuracy=\frac {1}{18}\sum _{j=1}^{18}\frac {T^{+}+T^{-}}{T^{+}+T^{-}+F^{+}+F^{-}}$, respectively. The accuracy, precision, sensitivity and specificity values of these three models and 18 different bacterial species have been listed in Table 1. From this statistical result, we can obviously see the accuracy and specificity of these neural networks can reach as high as 90%, while the precision and sensitivity are very low. The range of accuracy is from 0.912 to 0.997, reaching the high classification accuracy compared with that classified by naked eye. The high accuracy of classification for each kind of bacteria means these predicted catalogs are highly likely to be true, which verifies the feasibility of our three classification models. While the responding precision values are very low, from 0.080 to 0.960, meaning many bacterial colonies from the same species have certain possibility to be classified into different categories. The bacterial species responding to the lowest precision value is *Enterococcus faecalis*, which may be due to its lowest number of trained images. The lowest number of trained images will lead to insufficient level of learning colony features for description and discrimination, so that the precision and sensitivity can be affected. The misclassification may be generated from our sample variations, which may be introduced from bacteria living in different maconkey agar or blood agar. And it also means the repeatability of our models prediction is limited, maybe due to small number of neurons being integrated for classification. When the number of neurons for judgement is not enough, it will make the features representing for one bacterial species localized, which makes it hard to integrate enough feature weights to give a comprehensive judgement. In addition, these three models have many false negative values and few false positive ones which lead to overall low sensitivity and high specificity [25]. The range of sensitivity is from 0.069 to 1.000, and the species with lowest sensitivity value is *Enterococcus faecalis*, as excepted. However, the specificity of each bacteria is very high which can commonly reaches more than 0.950. This phenomenon may be because the number of neurons for giving bacterial category in fully connected layer is not enough, the screening process will be less strict to identify many false negative values. In this way, the not excluded false negative values will make the denominator bigger and the sensitivity lower. In another way, this less strict classification models can lead to lower false positive values and higher specificity. Thus they can serve their benefits of alerting clinical staffs to the situation when they classify some colonies into dangerous species.

**Table 1:** The statistical results of bacterial colony images classification using the three neural networks

	Accuracy			Precision			Sensitivity			Specificity		
	CNN	AlexNet	Autoencoder	CNN	AlexNet	Autoencoder	CNN	AlexNet	Autoencoder	CNN	AlexNet	Autoencoder
*Streptococcus agalactiae*	0.984	0.965	0.979	0.780	0.603	0.754	1.000	1.000	0.935	0.983	0.965	0.983
*Klebsiella oxytoca*	0.938	0.939	0.954	0.189	0.299	0.326	0.238	0.655	0.337	0.964	0.950	0.976
*Staphylococcus hominis*	0.997	0.993	0.989	0.938	0.881	0.773	0.900	0.740	0.680	0.999	0.998	0.996
*Stenotrophomonas maltophilia*	0.958	0.969	0.971	0.744	0.791	0.771	0.574	0.679	0.809	0.986	0.989	0.984
*Escherichia coli*	0.961	0.966	0.968	0.787	0.726	0.844	0.733	0.906	0.748	0.983	0.973	0.988
*Staphylococcus capitis*	0.988	0.986	0.981	0.722	0.626	0.609	0.839	1.000	0.677	0.992	0.986	0.989
*Proteus mirabilis*	0.938	0.974	0.951	0.659	0.870	0.683	0.315	0.722	0.567	0.987	0.993	0.981
*Enterococcus faecium*	0.981	0.912	0.969	0.783	0.172	0.361	0.310	0.776	0.373	0.998	0.916	0.984
*Burkholderia cepacia*	0.993	0.983	0.982	0.750	0.392	0.609	0.706	0.588	0.519	0.997	0.988	0.993
*Staphylococcus haemolyticus*	0.989	0.983	0.984	0.975	0.736	0.760	0.760	0.885	0.893	0.999	0.987	0.988
*Serratia marcescens*	0.981	0.922	0.977	0.844	0.889	0.735	0.663	0.248	0.649	0.995	0.997	0.991
*Enterococcus faecalis*	0.992	0.990	0.979	0.778	0.643	0.080	0.467	0.300	0.069	0.998	0.998	0.990
*Pseudomonas aeruginosa*	0.922	0.971	0.954	0.546	0.960	0.748	0.929	0.714	0.786	0.928	0.997	0.974
*Klebsiella pneumoniae*	0.955	0.983	0.964	0.678	0.942	0.733	0.520	0.760	0.707	0.984	0.997	0.985
*Staphylococcus epidermidis*	0.978	0.986	0.977	0.817	0.938	0.735	0.708	0.750	0.815	0.992	0.998	0.986
*Staphylococcus aureus*	0.972	0.983	0.975	0.845	0.959	0.923	0.958	0.901	0.878	0.977	0.995	0.990
*Enterococcus cloacae*	0.962	0.970	0.968	0.482	0.551	0.558	0.602	0.557	0.489	0.976	0.984	0.986
*Acinetobacter baumannii*	0.919	0.939	0.931	0.707	0.919	0.727	0.701	0.575	0.784	0.958	0.993	0.959
Average	0.967	0.967	0.970	0.723	0.716	0.652	0.662	0.709	0.651	0.983	0.984	0.985

### Comparison

To evaluate the feasibility of each deep learning based framework, the performance comparison was made among these three neural networks- conventional CNN, AlexNet and Autoencoder. According to accuracy and precision results, CNN and AlexNet methods are comparable, both having better precision performance than Autoencoder. This maybe because unsupervised neural network will extract some features that are not specific for bacterial classification, such as the features of agar background. For sensitivity and specificity, the two supervised networks also work better, and AlexNet has higher sensitivity than conventional CNN which maybe due to its highly compressed convolutional layers. Thus we can conclude that the supervised neural networks have more promising classification characteristics for bacterial colony application, and the unsupervised network should have more advantages in revealing novel characteristics from bacterial colony morphology [26].

### Clinical bacterial colony features

**Fig. 4:**
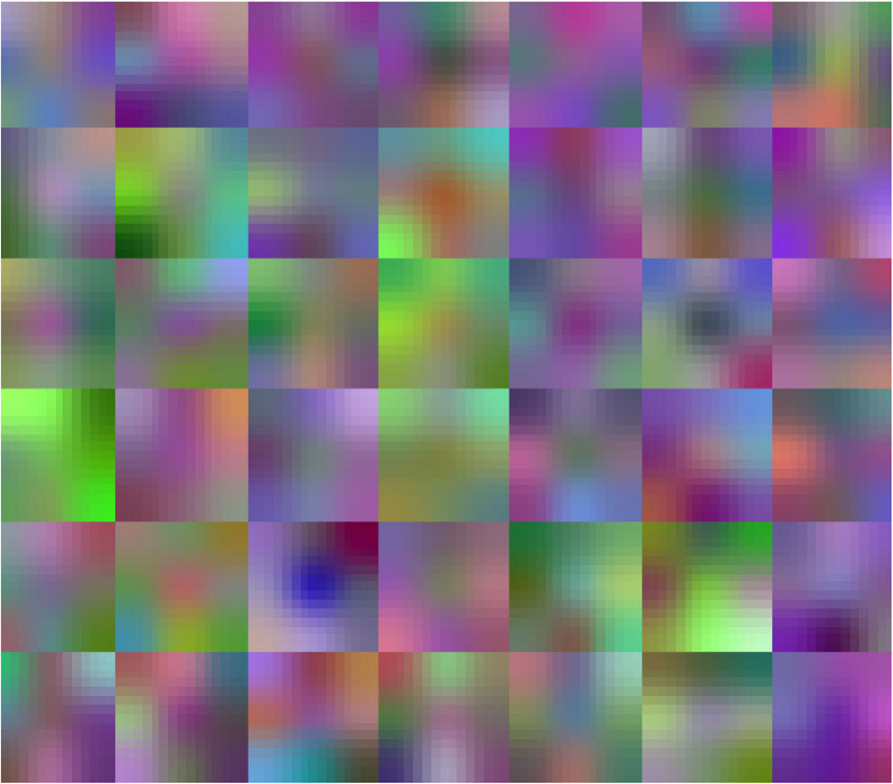
Bacterial colonies’ features extraction from the conventional convolutional neural network

**Fig. 5:**
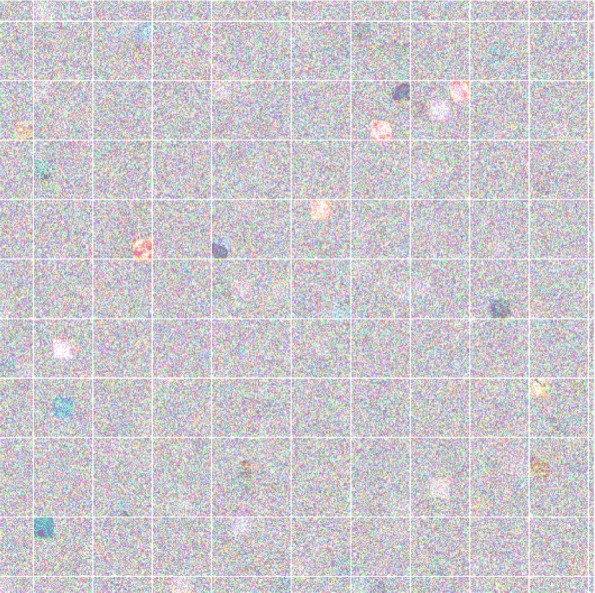
Bacterial colonies’ features extraction from the Autoencoder neural network

The Figs. 4 and 5 are the features learned from the conventional convolutional neural network and the Autoencoder neural network, respectively. It can be seen the features of the first layer are more related to the overall vague impression, like the stain-like texture. But the features from the Autoencoder neural network have some characteristics of shape and edge-like features [27]. This difference can be reasoned by whether this network training has been supervised or not. Because for clinical recognization, microbiologists are likely to distinguish different kinds of bacteria not only by their morphology of colonies, but also by considering the kind of culture media like the maconkey agar media or blood agar media, which have blue and red color background, respectively. Thus, the supervised convolutional neural network would be more likely to take the background color into consideration, and this recognized feature can be gotten from more than one round of backpropagation modification. On the other hand, the features extracted from the Autoencoder neural network will give us more intuitive discriminations about bacterial colonies, which seems that the shapes and the edge-like information of the bacterial colony are on the foreground of the extracted features that this neural network system is going to take into consideration. And we can learn some classification strategy from this for clinical bacterial colonies recognition.

### Performance evaluation of statistical results

Statistical results of classification of our unsupervised CNN are shown in Table 1. Because the unsupervised neural network is based on the natural morphologies of colonies’ features, we can learn some information about classification from them. There are different classification performances among different bacteria. The *Staphylococcus aureus* has the highest classification accuracy in the output class, while the *Klebsiella oxytoca* has the highest probability to misclassify. This may because *Klebsiella oxytoca* can grow in both maconkey and blood agar, and they can show different colony morphologies in the two different kinds of agar [28]. The samples we prepared for *Klebsiella oxytoca* includes both of these two morphologies from the same species, which makes the unsupervised network difficult to extract similar features from them, finally leading to low precision and sensitivity of classification.

### Sensitivity analysis

There are several issues needed to be considered about our three neural network models. The two critical conditions as the input values having objective influence on the final classification accuracy are sample size (n=4982) and the proportion of training set and testing set. The sample size of 4982 in our experiment is large, which means every species can have average 277 images for training and testing. However, not every bacterial species can have this number of images for simulation. In practice, we took photos in our clinical laboratory for several weeks to get all the data. Thus the number of images of each bacterial species was different from each other, which depended on the frequencies of detection in clinical laboratory in Peking University First Hospital. The more frequently the bacterial species was detected in clinic, the more number of images we can got, and more precise the classification method could be in theory. In this case, our classification method is very useful for practical application in clinic, because the more frequent-occurred species always means the more needs of attention in clinical diagnosis.

The following chart is the number of used images of each bacterial species. From this chart, we can see the numbers of images of *Burkholderia cepacia* and *Enterococcus faecalis* are less than 100, and both of them have relatively low sensitivity values, especially the *Enterococcus faecalis*. The number of available *Enterococcus faecalis* images is only 60, and the homogeneity of its colony is not very good, which are both related to its low sensitivity value. *Enterococcus faecalis* can have circle or elliptical colonies with blurry edges and the color is variable at different place, which may lead to the high probability of misclassification (Table 2).

**Table 2:** The number of images of each bacterial species

Bacterial species	Number of images
*Streptococcus agalactiae*	384
*Klebsiella oxytoca*	168
*Staphylococcus hominis*	100
*Stenotrophomonas maltophilia*	324
*Escherichia coli*	404
*Staphylococcus capitis*	124
*Proteus mirabilis*	352
*Enterococcus faecium*	118
*Burkholderia cepacia*	68
*Staphylococcus haemolyticus*	208
*Serratia marcescens*	196
*Enterococcus faecalis*	60
*Pseudomonas aeruginosa*	468
*Klebsiella pneumoniae*	300
*Staphylococcus epidermidis*	240
*Staphylococcus aureus*	624
*Enterococcus cloacae*	176
*Acinetobacter baumannii*	668
Total	4982

**Fig. 6:**
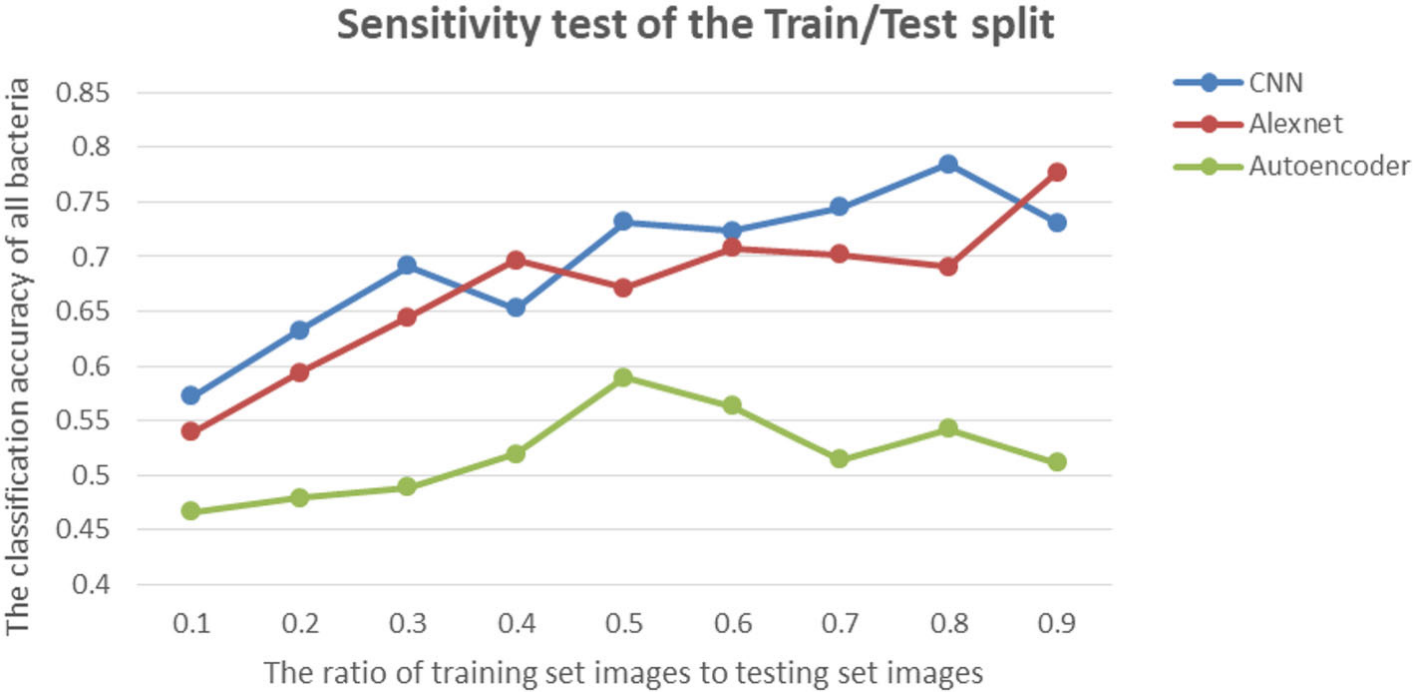
Sensitivity analysis of the Train/Test split

Another important input variable that may affect the training performance is the ratio of training set to testing set. As is described above, with certain number of available images, the low proportion of training set means small number of training images, which may lead to bad performance of feature description. To elucidate the effect of proportion changes on these three different neural networks, we made the sensitivity analysis. The ratio has been changed from 0.1 to 0.9, and the accuracy of all bacterial species has been calculated. The trend describing the correlation between the ratio and the total accuracy can be shown in Fig. 6.

From this figure we can clearly see the difference between supervised and unsupervised methods. The CNN and AlexNet neural networks can have an obvious increasing trend together with the ratio of training set to testing set. In contrast, the unsupervised Autoencoder method seems not have a significant relationship with the ratio changes. This may be because with the increasing number of training set images, the supervised neural networks can get more input data to learn more complicated information of specific features of each species, which is a process of data collection and can lead to their better performance. However, for Autoencoder, it does not depend on human experience but totally relies on its own feature extraction technique. The classification process will no longer be a process of data accumulation, thus the relationship between total accuracy and the ratio is not significant.

## Conclusion

This paper presented a computational bacterial colony classification system with three supervised and unsupervised neural networks. The detailed description about general structure, constitution of each layer and training processes are given in this paper. The parameters set in these neural networks could have different affections on the classification performance. The comparisons of classification performance among different neural networks are proposed, which can reflect the advantages of supervised convolutional neural networks over unsupervised ones. While for giving us more classification feature instructions, the unsupervised network is obviously a more dominant method, which can also provide some clinical bacterial colony features to our clinical staffs. And there are some advantages gotten from our computational vision classifier, which can help clinical staffs distinguish vague bacterial colonies without the use of manpower and clinical expertise, and the accuracy for each species of bacteria can reach as high as 90%. In addition, the low false positive values and high specificity of the predicted classification can serve as an alert to clinical staffs when some dangerous bacteria appear. Based on these points, our presented classification networks will have significant values referring to clinical experience and bacterial colony features.
